# Bis[2-(cyclo­hexyl­imino­meth­yl)-5-methoxy­phenolato]copper(II)

**DOI:** 10.1107/S1600536809051629

**Published:** 2009-12-04

**Authors:** Jian-Ying Miao

**Affiliations:** a Department of Chemistry and Chemical Engineering, Baoji University of Arts and Sciences, Baoji 721007, People’s Republic of China

## Abstract

In the title centrosymmetric mononuclear complex, [Cu(C_14_H_18_NO_2_)_2_], the Cu^II^ ion, lying on an inversion centre, is four-coordinated by two imine N and two phenolate O atoms from two Schiff base ligands, forming a slightly distorted square-planar geometry.

## Related literature

For general background to copper complexes, see: Collinson & Fenton (1996 ▶); Hossain *et al.* (1996 ▶); Tarafder *et al.* (2002 ▶); Musie *et al.* (2003 ▶); García-Raso *et al.* (2003 ▶); Reddy *et al.* (2000 ▶); Ray *et al.* (2003 ▶); Arnold *et al.* (2003 ▶); Raptopoulou *et al.* (1998 ▶). For related structures, see: Miao (2005 ▶, 2006 ▶); Wang (2007 ▶); Zhang (2004 ▶); Akitsu & Einaga (2004 ▶); Bluhm *et al.* (2003 ▶); Castillo *et al.* (2003 ▶); Lacroix *et al.* (2004 ▶).
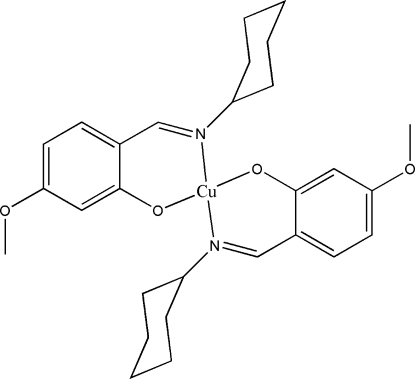


## Experimental

### 

#### Crystal data

[Cu(C_14_H_18_NO_2_)_2_]
*M* *_r_* = 528.13
Monoclinic, 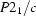

*a* = 6.4557 (10) Å
*b* = 11.5170 (17) Å
*c* = 17.074 (3) Å
β = 99.138 (2)°
*V* = 1253.4 (3) Å^3^
*Z* = 2
Mo *K*α radiation
μ = 0.91 mm^−1^
*T* = 298 K
0.23 × 0.20 × 0.20 mm

#### Data collection

Bruker SMART CCD area-detector diffractometer
Absorption correction: multi-scan (*SADABS*; Sheldrick, 1996 ▶) *T*_min_ = 0.818, *T*_max_ = 0.839
6860 measured reflections
2727 independent reflections
2232 reflections with *I* > 2σ(*I*)
*R*_int_ = 0.021

#### Refinement

*R*[*F* ^2^ > 2σ(*F* ^2^)] = 0.030
*wR*(*F* ^2^) = 0.080
*S* = 1.04
2727 reflections
161 parameters
H-atom parameters constrained
Δρ_max_ = 0.28 e Å^−3^
Δρ_min_ = −0.25 e Å^−3^

### 

Data collection: *SMART* (Bruker, 1998 ▶); cell refinement: *SAINT* (Bruker, 1998 ▶); data reduction: *SAINT*; program(s) used to solve structure: *SHELXS97* (Sheldrick, 2008 ▶); program(s) used to refine structure: *SHELXL97* (Sheldrick, 2008 ▶); molecular graphics: *SHELXTL* (Sheldrick, 2008 ▶); software used to prepare material for publication: *SHELXTL*.

## Supplementary Material

Crystal structure: contains datablocks global, I. DOI: 10.1107/S1600536809051629/ci2979sup1.cif

Structure factors: contains datablocks I. DOI: 10.1107/S1600536809051629/ci2979Isup2.hkl

Additional supplementary materials:  crystallographic information; 3D view; checkCIF report

## Figures and Tables

**Fig. 1.**
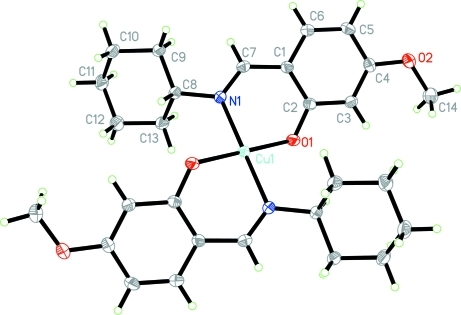
The molecular structure of the title compound, showing 30% displacement ellipsoids (arbitrary spheres for the H atoms). Unlabelled atoms are at the symmetry position (-x, -y, -z).

## supplementary crystallographic information

### Comment

In the last few years there has been a burgeoning effort to identify the biological activities of copper, primarily through techniques associated with the interface of biology/biochemistry/coordination chemistry (Collinson & Fenton, 1996; Hossain *et al.*, 1996; Tarafder *et al.*, 2002). It appears that the biological role of copper is primarily in redox reactions and as a biological catalyst, although much remains to be understood (Musie *et al.*, 2003; García-Raso *et al.*, 2003). An extensive effort has been made to prepare and characterize a variety of copper(II) coordination complexes in an attempt to model the physical and chemical behaviour of copper-containing enzymes (Reddy *et al.*, 2000). The peculiarity of copper lies in its ability to form complexes with coordination number four, five or six (Ray *et al.*, 2003; Arnold *et al.*, 2003; Raptopoulou *et al.*, 1998). As an extension of the work on the structural characterization of such complexes (Miao, 2005, 2006), the crystal structure of the title new mononuclear copper(II) compound, is reported here.

The compound is a centrosymmetric mononuclear copper(II) complex, as shown in Fig. 1. The Cu^II^ ion, lying on an inversion centre, is four-coordinated by two imine N and two phenolate O atoms from two Schiff base ligands, forming a square-planar geometry. The Cu—O and Cu—N bond lengths are comparable with those reported in similar structures (Wang, 2007; Zhang, 2004; Akitsu & Einaga, 2004; Bluhm *et al.*, 2003; Castillo *et al.*, 2003; Lacroix *et al.*, 2004). Both cyclohexane rings adopt chair conformations.

### Experimental

4-Methoxysalicylaldehyde (1 mmol, 152 mg), cyclohexylamine (1 mmol, 99 mg) and Cu(CH_3_COO)_2_.H_2_O (0.5 mmol, 100 mg) were dissolved in methanol (50 ml). The mixture was stirred at room temperature for 1 h to give a blue solution. The resulting solution was kept in air for 5 d, and block blue crystals were formed.

### Refinement

H atoms were placed in idealized positions and constrained to ride on their parent atoms, with C—H distances in the range 0.93–0.98 Å, and with *U*_iso_(H) = 1.2 or 1.5*U*_eq_(C).

### Crystal data

**Table:**

[Cu(C_14_H_18_NO_2_)_2_]	*F*(000) = 558
*M**_r_* = 528.13	*D*_x_ = 1.399 Mg m^−^^3^
Monoclinic, *P*2_1_/*c*	Mo *K*α radiation, λ = 0.71073 Å
Hall symbol: -P 2ybc	Cell parameters from 2706 reflections
*a* = 6.4557 (10) Å	θ = 2.4–28.7°
*b* = 11.5170 (17) Å	µ = 0.91 mm^−^^1^
*c* = 17.074 (3) Å	*T* = 298 K
β = 99.138 (2)°	Block, blue
*V* = 1253.4 (3) Å^3^	0.23 × 0.20 × 0.20 mm
*Z* = 2	

### Data collection

**Table:**

Bruker SMART CCD area-detector diffractometer	2727 independent reflections
Radiation source: fine-focus sealed tube	2232 reflections with *I* > 2σ(*I*)
graphite	*R*_int_ = 0.021
ω scans	θ_max_ = 27.0°, θ_min_ = 2.1°
Absorption correction: multi-scan (*SADABS*; Sheldrick, 1996)	*h* = −6→8
*T*_min_ = 0.818, *T*_max_ = 0.839	*k* = −14→14
6860 measured reflections	*l* = −17→21

### Refinement

**Table:**

Refinement on *F*^2^	Primary atom site location: structure-invariant direct methods
Least-squares matrix: full	Secondary atom site location: difference Fourier map
*R*[*F*^2^ > 2σ(*F*^2^)] = 0.030	Hydrogen site location: inferred from neighbouring sites
*wR*(*F*^2^) = 0.080	H-atom parameters constrained
*S* = 1.03	*w* = 1/[σ^2^(*F*_o_^2^) + (0.0393*P*)^2^ + 0.3443*P*] where *P* = (*F*_o_^2^ + 2*F*_c_^2^)/3
2727 reflections	(Δ/σ)_max_ = 0.001
161 parameters	Δρ_max_ = 0.28 e Å^−^^3^
0 restraints	Δρ_min_ = −0.25 e Å^−^^3^

### Special details

**Table:**

Geometry. All e.s.d.'s (except the e.s.d. in the dihedral angle between two l.s. planes) are estimated using the full covariance matrix. The cell e.s.d.'s are taken into account individually in the estimation of e.s.d.'s in distances, angles and torsion angles; correlations between e.s.d.'s in cell parameters are only used when they are defined by crystal symmetry. An approximate (isotropic) treatment of cell e.s.d.'s is used for estimating e.s.d.'s involving l.s. planes.
Refinement. Refinement of *F*^2^ against ALL reflections. The weighted *R*-factor *wR* and goodness of fit *S* are based on *F*^2^, conventional *R*-factors *R* are based on *F*, with *F* set to zero for negative *F*^2^. The threshold expression of *F*^2^ > σ(*F*^2^) is used only for calculating *R*-factors(gt) *etc*. and is not relevant to the choice of reflections for refinement. *R*-factors based on *F*^2^ are statistically about twice as large as those based on *F*, and *R*- factors based on ALL data will be even larger.

### Fractional atomic coordinates and isotropic or equivalent isotropic displacement parameters (Å^2^)

**Table:**

	*x*	*y*	*z*	*U*_iso_*/*U*_eq_	
Cu1	0.0000	0.0000	0.0000	0.02741 (11)	
N1	0.2825 (2)	−0.05099 (13)	0.05759 (8)	0.0279 (3)	
O1	−0.02292 (18)	0.11030 (12)	0.08083 (8)	0.0369 (3)	
O2	0.2183 (2)	0.44832 (13)	0.22866 (8)	0.0435 (3)	
C1	0.3444 (3)	0.13149 (15)	0.12935 (10)	0.0293 (4)	
C2	0.1333 (3)	0.17066 (15)	0.11880 (10)	0.0290 (4)	
C3	0.0893 (3)	0.27716 (16)	0.15350 (11)	0.0324 (4)	
H3	−0.0488	0.3025	0.1492	0.039*	
C4	0.2490 (3)	0.34426 (15)	0.19372 (10)	0.0321 (4)	
C5	0.4579 (3)	0.30770 (17)	0.20160 (12)	0.0377 (4)	
H5	0.5654	0.3543	0.2271	0.045*	
C6	0.5013 (3)	0.20248 (16)	0.17120 (11)	0.0350 (4)	
H6	0.6396	0.1769	0.1784	0.042*	
C7	0.4013 (3)	0.01873 (15)	0.10362 (11)	0.0314 (4)	
H7	0.5373	−0.0064	0.1220	0.038*	
C8	0.3570 (3)	−0.17037 (15)	0.04404 (11)	0.0292 (4)	
H8	0.3409	−0.1818	−0.0135	0.035*	
C9	0.5849 (3)	−0.19822 (16)	0.07803 (12)	0.0344 (4)	
H9A	0.6775	−0.1459	0.0555	0.041*	
H9B	0.6075	−0.1871	0.1351	0.041*	
C10	0.6355 (3)	−0.32350 (17)	0.05889 (14)	0.0425 (5)	
H10A	0.7792	−0.3409	0.0820	0.051*	
H10B	0.6229	−0.3327	0.0019	0.051*	
C11	0.4882 (3)	−0.40877 (17)	0.09089 (14)	0.0448 (5)	
H11A	0.5208	−0.4873	0.0763	0.054*	
H11B	0.5082	−0.4041	0.1483	0.054*	
C12	0.2621 (3)	−0.38098 (17)	0.05739 (14)	0.0455 (5)	
H12A	0.1699	−0.4333	0.0801	0.055*	
H12B	0.2392	−0.3927	0.0004	0.055*	
C13	0.2090 (3)	−0.25579 (16)	0.07562 (13)	0.0389 (4)	
H13A	0.2194	−0.2461	0.1325	0.047*	
H13B	0.0656	−0.2391	0.0517	0.047*	
C14	0.0085 (4)	0.48443 (19)	0.23134 (17)	0.0591 (7)	
H14A	−0.0676	0.4892	0.1784	0.089*	
H14B	0.0100	0.5592	0.2562	0.089*	
H14C	−0.0583	0.4292	0.2612	0.089*	

### Atomic displacement parameters (Å^2^)

**Table:**

	*U*^11^	*U*^22^	*U*^33^	*U*^12^	*U*^13^	*U*^23^
Cu1	0.02394 (16)	0.02709 (17)	0.02975 (17)	0.00081 (12)	−0.00020 (11)	−0.00354 (12)
N1	0.0262 (7)	0.0262 (7)	0.0306 (8)	0.0021 (6)	0.0025 (6)	0.0001 (6)
O1	0.0262 (6)	0.0415 (7)	0.0409 (7)	0.0005 (5)	−0.0011 (5)	−0.0141 (6)
O2	0.0426 (8)	0.0368 (8)	0.0501 (8)	−0.0028 (6)	0.0043 (6)	−0.0145 (7)
C1	0.0277 (9)	0.0301 (9)	0.0289 (9)	−0.0006 (7)	0.0011 (7)	−0.0003 (7)
C2	0.0281 (9)	0.0321 (9)	0.0260 (8)	−0.0018 (7)	0.0018 (7)	0.0001 (7)
C3	0.0274 (9)	0.0356 (10)	0.0335 (9)	0.0023 (7)	0.0024 (7)	−0.0039 (8)
C4	0.0396 (10)	0.0278 (9)	0.0287 (9)	−0.0018 (8)	0.0045 (8)	−0.0017 (7)
C5	0.0327 (10)	0.0374 (11)	0.0412 (11)	−0.0090 (8)	−0.0001 (8)	−0.0053 (9)
C6	0.0269 (9)	0.0371 (10)	0.0393 (10)	−0.0012 (8)	0.0005 (7)	−0.0015 (8)
C7	0.0251 (9)	0.0355 (10)	0.0325 (9)	0.0015 (7)	0.0014 (7)	0.0025 (7)
C8	0.0275 (9)	0.0278 (9)	0.0322 (9)	0.0027 (7)	0.0040 (7)	0.0009 (7)
C9	0.0280 (9)	0.0311 (10)	0.0439 (11)	0.0013 (7)	0.0047 (8)	0.0026 (8)
C10	0.0333 (10)	0.0361 (11)	0.0592 (13)	0.0076 (8)	0.0107 (9)	0.0050 (9)
C11	0.0474 (12)	0.0291 (10)	0.0588 (13)	0.0049 (9)	0.0115 (10)	0.0069 (9)
C12	0.0433 (11)	0.0317 (10)	0.0634 (14)	−0.0039 (9)	0.0143 (10)	0.0029 (10)
C13	0.0297 (9)	0.0347 (10)	0.0537 (12)	−0.0009 (8)	0.0111 (8)	0.0024 (9)
C14	0.0502 (13)	0.0549 (15)	0.0724 (17)	0.0048 (11)	0.0106 (12)	−0.0306 (12)

### Geometric parameters (Å, °)

**Table:**

Cu1—O1^i^	1.8987 (12)	C8—C9	1.527 (2)
Cu1—O1	1.8987 (12)	C8—C13	1.528 (2)
Cu1—N1^i^	2.0169 (14)	C8—H8	0.98
Cu1—N1	2.0169 (14)	C9—C10	1.526 (3)
N1—C7	1.288 (2)	C9—H9A	0.97
N1—C8	1.487 (2)	C9—H9B	0.97
O1—C2	1.309 (2)	C10—C11	1.527 (3)
O2—C4	1.367 (2)	C10—H10A	0.97
O2—C14	1.424 (3)	C10—H10B	0.97
C1—C6	1.406 (2)	C11—C12	1.515 (3)
C1—C2	1.420 (2)	C11—H11A	0.97
C1—C7	1.437 (2)	C11—H11B	0.97
C2—C3	1.411 (2)	C12—C13	1.525 (3)
C3—C4	1.381 (2)	C12—H12A	0.97
C3—H3	0.93	C12—H12B	0.97
C4—C5	1.399 (3)	C13—H13A	0.97
C5—C6	1.365 (3)	C13—H13B	0.97
C5—H5	0.93	C14—H14A	0.96
C6—H6	0.93	C14—H14B	0.96
C7—H7	0.93	C14—H14C	0.96
			
O1^i^—Cu1—O1	180.00 (10)	C13—C8—H8	107.1
O1^i^—Cu1—N1^i^	90.53 (5)	C10—C9—C8	110.06 (15)
O1—Cu1—N1^i^	89.47 (5)	C10—C9—H9A	109.6
O1^i^—Cu1—N1	89.47 (5)	C8—C9—H9A	109.6
O1—Cu1—N1	90.53 (5)	C10—C9—H9B	109.6
N1^i^—Cu1—N1	180.00 (11)	C8—C9—H9B	109.6
C7—N1—C8	119.69 (15)	H9A—C9—H9B	108.2
C7—N1—Cu1	121.37 (12)	C9—C10—C11	111.40 (16)
C8—N1—Cu1	118.90 (11)	C9—C10—H10A	109.3
C2—O1—Cu1	124.93 (11)	C11—C10—H10A	109.3
C4—O2—C14	118.32 (15)	C9—C10—H10B	109.3
C6—C1—C2	118.65 (16)	C11—C10—H10B	109.3
C6—C1—C7	118.84 (16)	H10A—C10—H10B	108.0
C2—C1—C7	122.36 (16)	C12—C11—C10	110.27 (17)
O1—C2—C3	118.64 (15)	C12—C11—H11A	109.6
O1—C2—C1	122.87 (16)	C10—C11—H11A	109.6
C3—C2—C1	118.44 (16)	C12—C11—H11B	109.6
C4—C3—C2	120.76 (16)	C10—C11—H11B	109.6
C4—C3—H3	119.6	H11A—C11—H11B	108.1
C2—C3—H3	119.6	C11—C12—C13	110.90 (17)
O2—C4—C3	124.00 (17)	C11—C12—H12A	109.5
O2—C4—C5	115.23 (16)	C13—C12—H12A	109.5
C3—C4—C5	120.77 (17)	C11—C12—H12B	109.5
C6—C5—C4	118.93 (17)	C13—C12—H12B	109.5
C6—C5—H5	120.5	H12A—C12—H12B	108.0
C4—C5—H5	120.5	C12—C13—C8	111.31 (15)
C5—C6—C1	122.33 (17)	C12—C13—H13A	109.4
C5—C6—H6	118.8	C8—C13—H13A	109.4
C1—C6—H6	118.8	C12—C13—H13B	109.4
N1—C7—C1	126.40 (17)	C8—C13—H13B	109.4
N1—C7—H7	116.8	H13A—C13—H13B	108.0
C1—C7—H7	116.8	O2—C14—H14A	109.5
N1—C8—C9	116.81 (14)	O2—C14—H14B	109.5
N1—C8—C13	107.74 (13)	H14A—C14—H14B	109.5
C9—C8—C13	110.43 (15)	O2—C14—H14C	109.5
N1—C8—H8	107.1	H14A—C14—H14C	109.5
C9—C8—H8	107.1	H14B—C14—H14C	109.5

Symmetry codes: (i) −*x*, −*y*, −*z*.
